# Risk factors of lateral lymph node metastasis in cN0 papillary thyroid carcinoma

**DOI:** 10.1186/s12957-018-1336-3

**Published:** 2018-02-13

**Authors:** Daixing Hu, Jing Zhou, Wei He, Jie Peng, Yijia Cao, Haoyu Ren, Yu Mao, Yi Dou, Wei Xiong, Qi Xiao, Xinliang Su

**Affiliations:** Department of Endocrine and Breast Surgery, The First Affiliated Hospital of Chongqing Medical University, Full address: 1 Friendship Road, Yu Zhong District, Chongqing, 400016 China

**Keywords:** Papillary thyroid carcinoma, Central lymph node dissection, Lateral lymph node dissection, Clinical lymph node negative

## Abstract

**Background:**

Cervical lymph node metastasis of papillary thyroid carcinoma (PTC) is common. However, whether undergoing prophylactic central lymph node (CLN) dissection or lateral lymph node (LLN) dissections to prevent metastasis is still controversial. This study aimed to retrospectively investigate the risk factors of LLN metastasis in clinical lymph node-negative (cN0) PTC patients.

**Methods:**

We retrospectively studied 783 lymph node-negative (cN0) PTC patients who underwent total thyroidectomy plus CLN dissection and LLN dissection.

**Results:**

The rates of CLN and LLN metastases were 68.2 and 47.4%, respectively. Large tumor size (> 20 mm) had a fourfold higher risk of LLN metastasis compared with small tumor size (≤ 20 mm; OR = 4.082, 95% CI 2.646–6.289; *P* = 0.001). Patients with tumor in the upper lobe had ~ 3-fold higher risk of LLN metastasis compared with patients with tumor in other locations (OR = 2.874, 95% CI 1.916–4.310; *P* = 0.001). Multifocality and extrathyroidal extension indicated a twofold higher risk of LLN metastasis. Having ≥ 2 CLN metastases dramatically increased the risk of LLN metastasis, compared with those with < 2 CLN metastases (OR = 6.536, 95% CI 4.630–9.259; *P* = 0.001).

**Conclusions:**

Large tumor size (> 20 mm), tumor located in the upper lobe, multifocality, extrathyroidal extension, and ≥ 2 CLN metastases may increase the risk of LLN metastasis in cN0 PTC patients.

## Background

Papillary thyroid carcinoma (PTC) is a common endocrine malignancy [1] and the most common type of thyroid cancer. In the USA, the annual incidence of PTC has increased during the last few decades, from 3.4/100,000 persons in 1975 to 12.5/100,000 persons in 2009 [2]. The etiology of PTC may associate with factors such as iodine deficiency, history of radiation on neck, Hashimoto’s thyroiditis, hereditary factors, and BRAF mutation [3, 4]. However, the increasing incidence cannot be fully explained by these risk factors. The majority of PTC patients have an excellent prognosis with low mortality rate, but early-stage metastasis may occur in the cervical lymph nodes.

Approximately 20–90% of PTC patients developed lymph node metastasis according to previous studies [5, 6]. These studies also indicate that lymph node metastasis predicts a higher recurrence rate. However, lymph node metastasis may not be associated with overall survival in PTC patients [7]. Therefore, the surgical treatment for metastasis of PTC remains controversial, especially the application of total thyroidectomy or lobectomy [8], and the prophylactic dissections of central and lateral lymph nodes (CLN and LLN, respectively) are also debatable [9].

In this retrospective cross-sectional study, we investigated the risk factors of LLN metastasis in clinical lymph node-negative (cN0) PTC patients.

## Methods

**Fig. 1:**
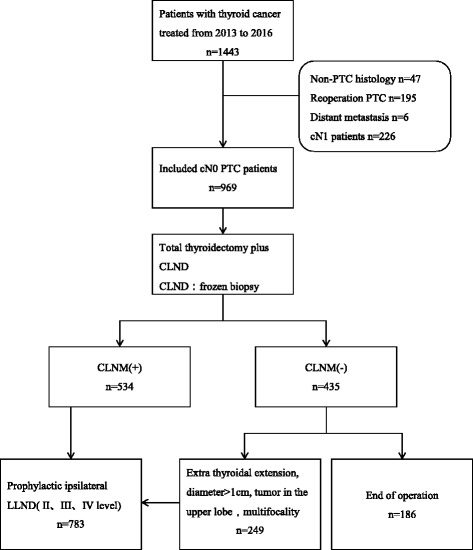
Flowchart of this retrospective study. CLND, central lymph node dissection; CLNM, central lymph node metastasis; LLND, lateral lymph node dissection

A total of 1443 patients underwent surgery for thyroid cancer at the Department of Endocrine and Breast Surgery, First Affiliated Hospital of Chongqing Medical University, between January 2013 and December 2016. Patients with any of the following were excluded: non-PTC histology (*n* = 47), reoperation (*n* = 195), distant metastasis (*n* = 6), and clinical lymph node-positive (cN1) patients (*n* = 226). The inclusion criteria were as follows: no history of head or neck irradiation, and clinical lymph node-negative (cN0) confirmed by palpation and neck ultrasound. A total of 969 cN0 PTC patients underwent total thyroidectomy with CLN dissection and with or without LLN dissection. Routine ipsilateral central lymph node frozen biopsy is performed. Prophylactic LLN dissection was performed if CLN metastasis was confirmed by frozen biopsy. Because of the long-term clinical observation, some CLN-negative PTC patients diagnosed by intraoperative frozen biopsy, who still underwent prophylactic lateral lymph node dissection, meet the following conditions: extra thyroidal extension, diameter > 1 cm, tumor in the upper lobe, and multifocality (Fig. 1).

We sent all of the central compartment tissue for frozen biopsy after CLN dissection, and then, experienced pathologists dissected CLN from the fat tissue and examined the lymph nodes specimens. The frozen biopsy diagnosis takes about 30 min. All harvested lymph nodes were sent to postoperative pathological examination according to different regions and were diagnosed by three pathologists.

In our institution, total thyroidectomy plus ipsilateral CLN dissection or ipsilateral LLN dissection is performed for unilateral PTC with a diameter > 1cm. For patients who have bilateral thyroid cancer with diameter > 1 cm, total thyroidectomy plus bilateral CLN dissection and bilateral LLN dissection is performed. For PTC with diameter <1 cm, lobectomy with ipsilateral CLN dissection is the basic operative treatment. If the ipsilateral central lymph node frozen biopsy suggests lymph node metastasis, we further perform contralateral lobectomy plus ipsilateral LLN dissection. The grading of each LLN level is based on the guidelines of the American Head and Neck Society [10]. Level I and level V lymph node metastases are rare in clinic, as previously reported [11, 12]. We performed selective LLN dissections, and the main regions of the LLN were graded as II, III, or IV.

Demographic and clinical pathology data were collected, including gender, age, extrathyroidal extension (ETE), multifocality, Hashimoto’s thyroiditis, tumor size, tumor location, bilaterality, and number of central lymph node metastases. If the patient had multiple lesions in one side of the thyroid gland, the diameter of the largest was recorded as the tumor size.

Postoperative complications were also evaluated and mainly concerned hemorrhage, hypoparathyroidism, recurrent laryngeal nerve injury, or chyle leakage. Serum calcium < 8 mg/dL within 6 months after surgery was defined as temporary hypoparathyroidism, while after 6 months was regarded as permanent hypoparathyroidism. Vocal cord paralysis, found by laryngoscopy within 6 months after the operation, was defined as temporary recurrent laryngeal nerve injury; vocal cord paralysis that lasted longer than 6 months was defined as permanent laryngeal nerve injury.

Recurrence can be seen as local or regional disease requiring reoperation or other treatment 6 months after the initial standard operation. It is diagnosed by ultrasound or ultrasound-guided fine needle aspiration cytology.

### Statistical analysis

*T* test, chi-square, or Fisher’s exact test were used to compare differences in gender (male, female), age (≤ 45, > 45), tumor size (≤ 10, 10–20, > 20 mm), tumor location (upper lobe, middle lobe, lower lobe, isthmus, hole), ETE (yes, no), multifocality (yes, no), bilaterality (yes, no), Hashimoto’s thyroiditis (yes, no), and number of central lymph node metastases (≤ 1, ≥ 2) between the patients with or without LLN metastases. Univariate and multivariate analyses were used in this study. Two-sided *P* < 0.05 was regarded as statistically significant. SPSS version 21 software was used for all analyses. The study was approved by the local institutional ethics committee board.

## Results

There were 783 PTC patients who underwent total thyroidectomy, plus prophylactic CLN dissection and LLN dissection from January 2013 to December 2016. All patients underwent ultrasonography, fibrolaryngoscope, and thyroid function before surgery. The mean follow-up time was 20.2 ± 12.7 months.

The total study population consisted of 576 (73.6%) women and 207 (26.4%) men (Table 1). The patient group with LLN metastasis comprised 256 women and 115 men; the group without LLN metastasis consisted of 320 women and 92 men (*P* = 0.06). The average age at diagnosis of the metastasis group was 41.0 ± 13.3, lower than that of the group without metastasis (*P* = 0.005). The tumor size in the metastasis group (20.8 ± 12.2 mm) was significantly larger than that of the patients without metastasis (14.0 ± 9.0 mm; *P* = 0.001). In the metastasis group, 105 (28.3%) patients had tumor in the upper lobe, while in the group without metastasis, this was true in only 68 (16.5%). The incidence of ETE, bilateral lesions, were also different between the two groups (all *P* < 0.05).

**Table 1:** Baseline characteristics of the study patients

			With LLN metastasis		
		Total	Yes	No	*P*
Subjects, *n*		783	371	412	–
Gender	Male	207 (26.4)	115 (31.0)	92 (22.3)	0.006
	Female	576 (73.6)	256 (69.0)	320 (77.7)	
Age at diagnosis, years		42.4 ± 13.1	41.0 ± 13.7	43.8 ± 12.3	0.005
Age group, years	≤ 45	473 (60.0)	242 (65.2)	231 (56.1)	0.009
	> 45	310 (40.0)	129 (34.8)	181 (43.9)	
Tumor size, mm		17.2 ± 11.1	20.8 ± 12.2	14.0 ± 9.0	0.001
Tumor size group	≤ 10	306 (39.1)	94 (25.3)	212 (51.5)	0.001
	10–20	315 (40.0)	161 (43.4)	154 (37.4)	
	> 20	162 (20.9)	116 (31.3)	46 (11.1)	
Tumor location	Upper lobe	173 (22.1)	105 (28.3)	68 (16.5)	0.001^a^
	Middle lobe	345 (44.1)	155 (41.8)	190 (46.1)	
	Lower lobe	217 (27.7)	75 (20.2)	142 (34.4)	
	Isthmus	19 (2.4)	11 (3.0)	8 (2.0)	
	Whole	29 (3.7)	25 (6.7)	4 (1.0)	
ETE	Yes	157 (20.1)	96 (25.9)	61 (14.8)	0.001
	No	626 (79.9)	275 (74.1)	351 (85.2)	
Multifocality	Yes	94 (12.0)	54 (14.6)	40 (9.7)	0.037
	No	689 (88.0)	317 (85.4)	372 (90.3)	
Bilaterality	Yes	131 (16.7)	75 (20.2)	56 (13.6)	0.013
	No	652 (83.3)	296 (79.8)	356 (86.4)	
Hashimoto’s thyroiditis	Yes	92 (11.7)	46 (12.4)	46 (11.2)	0.592
	No	691 (88.3)	325 (87.6)	366 (88.8)	
CLN metastases	< 2	383 (48.9)	96 (25.9)	287 (69.7)	0.001
	≥ 2	400 (51.1)	275 (74.1)	125 (30.3)	

Reported as *n* (%), unless noted otherwise. *P* values represent the statistically difference between the groups with and without LLN metastasis, unless noted otherwise

^a^The *P* value means the difference among the upper lobe, middle lobe, lower lobe, isthmus, and hole thyroid lobe

LLN metastasis was significantly associated with gender, age at diagnosis, age group (≤ 45, > 45 years), tumor size, tumor size group, tumor location, ETE, multifocality, bilateral, and number of CLN metastasis in the univariate analysis (Table 1). In the multivariate analysis, compared to the women, the men tended to have a higher risk of LLN metastasis (OR = 1.435, 95% CI 0.982–2.092), although no statistical significance was found (*P* = 0.062; Table 2). No risk differences in LLN metastasis were found between the age groups (*P* = 0.373) and bilateral groups (*P* = 0.866). Large tumor size (> 20 mm) was observed with a fourfold higher risk of LLN metastasis compared with small tumor size (≤ 20 mm; OR = 4.082, 95% CI 2.646–6.289; *P* = 0.001). Patients with tumor found in the upper lobe had ~ 3-fold higher risk of LLN metastasis compared with patients with tumor in other locations (OR = 2.874, 95% CI 1.916–4.310*; P* = 0.001). Multifocality and ETE indicated a twofold higher risk of LLN metastasis. Having > 2 CLN metastases dramatically increased the risk of LLN metastasis, compared with those with < 2 CLN metastasis (OR = 6.536, 95% CI 4.630–9.259; *P* = 0.001).

**Table 2:** Multivariate analysis of risk factors of LLN metastasis

	OR	95% CI	*P value*
Gender (male vs. female)	1.435	0.982–2.092	0.062
Age (≤ 45 vs. > 45 years)	1.171	0.827–1.658	0.373
Tumor size (> 20 mm vs. ≤ 20 mm)	4.082	2.646–6.289	0.001
Tumor location (upper lobe vs. non-upper lobe)	2.874	1.916–4.310	0.001
Bilaterality (yes vs. no)	1.041	0.657–1.647	0.866
Multifocality (yes vs. no)	1.832	1.083–3.096	0.024
Extra thyroidal extension (yes vs. no)	1.838	1.208–2.801	0.005
Number of CLN metastasis (< 2 vs. ≤ 1)	6.536	4.630–9.259	0.001

Among the 783 PTC patients, only two cases of hemorrhage were found (Table 3). Temporary hypoparathyroidism was the most frequent comorbidity observed in both the metastasis and non-metastasis groups, with slightly more cases in the former (*P* = 0.019). Chyle leakage occurred more frequently in the patients with LLN metastasis (*P* = 0.031). No other differences were found regarding the occurrence of comorbidity. Five patients experienced recurrence, 4 of which were in the metastasis-positive group.

**Table 3:** Comorbidity and recurrence of LLN metastasis

	LLN metastasis		*P* value
	Yes	No	
Subjects, *n*	371	412	–
Hemorrhage	2	0	0.136
Temporary hypoparathyroidism	214	203	0.019
Permanent hypoparathyroidism	2	1	0.503
Temporary recurrent laryngeal nerve injury	16	14	0.506
Permanent recurrent laryngeal nerve injury	2	2	0.916
Chyle leakage	10	3	0.031
Recurrence	4	1	0.134

## Discussion

Our study indicates that large tumor size, tumor in the upper lobe, multifocality, ETE, and having ≥ 2 CLN metastases were all associated positively with risk of LLN metastasis. Gender, age, and bilateral lesions were not associated with risk of LLN metastasis.

The univariate analysis showed that the risk of LLN metastasis was significantly higher in men than in women, but this association did not hold true in the multivariate analysis. Previous studies found that male gender was associated with CLN metastasis, but was not associated with LLN metastasis [13, 14]. In the present study, age was not associated with risk of LLN metastasis, which is in agreement with two previous studies [13, 15].

Hunt et al. [16] suggested that the location of the tumor is closely related to LLN metastasis. More specifically, Zhang et al. [17] found that a tumor in the upper third of the thyroid was at greater risk of LLN metastasis, but at lower risk of CLN metastasis. Kwak et al. [18] also demonstrated that an upper pole location was an independent factor in predicting LLN metastasis.

Many studies have shown that lymph node metastasis is associated with tumor size. Nie et al. [15] suggested that a tumor diameter > 1.5 cm is an independent risk factor for LLN metastasis. However, another study suggested a cut-off point of 2 cm [13], which is in accord with the results of the current study.

In our study, multifocality was found to increase the risk of LLN metastasis. However, bilateral lesions were not a potential risk factor. Nie et al. [15] suggested that multifocality and bilateral were not risk factors for lymph node metastasis.

Much evidence has indicated that ETE is an important predictor of lymph node metastasis in PTC patients [19, 20]. Previous research has shown that CLN metastasis is an important risk factor for LLN metastasis [21, 22]. More specifically, our present study found that patients with ≥ 2 CLN metastases had higher risk of LLN metastasis, which could potentially provide support for performing LLN dissection.

Five cases of recurrence were found in the present study. Every patient had central lymph node and four patients had LLN metastasis during the initial treatment. Mercante et al. [23] found that lymph node metastasis was a risk factor of local recurrence. Chereau et al. [24] reported the LLN metastasis is an important prognostic factor for recurrence. Five patients were treated with iodine 131 after the initial operation. The average time of recurrence was 22.4 months (range from 12 to 35 months). In the current study, recurrence was in the LLN of each patient, which draws our particular attention to the question of LLN dissection.

Surgical complications are so important that they cannot be ignored. Common complications include parathyroid injury, recurrent laryngeal nerve, chyle leakage, and Horner’s syndrome. Moo et al. [25] reported that rates of temporary and permanent hypocalcemia were 47 and 0%, respectively, and the rates for temporary and permanent recurrent laryngeal nerve injury were 5 and 0%. Our data showed that the incidence of temporary parathyroid injury was high (417/783; 53.2%), but most patients had parathyroid function recovery after symptomatic treatment. There were very few patients with permanent parathyroid injury or permanent recurrent laryngeal nerve injury. Thirteen patients had chyle leakage, and each patient underwent level IV lymph node dissection; 11 of these underwent left level IV lymph node dissection. Twelve patients were treated successfully by strong negative pressure drainage, low-fat diet, or intracavitary injection of inactivated *Pseudomonas aeruginosa*. Only one patient underwent reoperation for thoracic duct ligation. We should pay special attention to left level IV lymph node dissection, as it may damage the branch or trunk of the thoracic duct because of its anatomical position.

We have gone through the process from roughly zoning and wide dissection to precise zoning and selective dissection. In our study, the recommended procedure for PTC patients is lobectomy with paratracheal, prelaryngeal, and ipsilateral tracheal lymph node dissections (we recommend posterior lymph nodes of recurrent laryngeal nerve dissection, if the lesions are located on the right side of the lobe). Then, the abovementioned central lymph node will undergo an intraoperative frozen biopsy, because the number of ipsilateral CLN metastases is very important.

If the total number of ipsilateral central metastases is ≥ 2, then prophylactic LLN dissection mainly in the ipsilateral level III and IV should be performed. Preoperative ultrasound showing enlarged lymph nodes in level II, intraoperative frozen biopsy prelaryngeal lymph node metastasis, and a primary tumor located in the upper pole of the thyroid gland is the basis of level II lymph node dissection.

## Conclusions

In conclusion, our study found that large tumor size, tumor in the upper lobe, multifocality, ETE, or ≥ 2 CLN metastases may increase the risk of LLN metastasis. Recurrence was high among patients with LLN metastasis, and therefore, we support that PTC patients need routine central lymph node dissection, not only for more accurate clinical staging but also because of the high incidence of central lymph node metastasis.

We recommend that prophylactic LLN dissection should be considered for PTC patients with large tumor size (> 20 mm), primary tumor in the upper lobe, multifocality, ETE, or ≥ 2 CLN metastases.

## Abbreviations

CLN: Central lymph node
cN0: Clinical lymph node negative
LLN: Lateral lymph node
PTC: Papillary thyroid carcinoma
